# Shear Bond Strength of Ceramic Bonded to Different Core Materials and Their Pattern of Failure: An In Vitro Study

**DOI:** 10.7759/cureus.6242

**Published:** 2019-11-26

**Authors:** Shilpa P, Narendra R, Sesha Reddy, Sashideepth Reddy

**Affiliations:** 1 Prosthodontics, Government Dental College and Hospital, Kadapa, IND

**Keywords:** bond strength, ceramic bonded core

## Abstract

Introduction

In metal-ceramic restoration, most of the bond failures between the ceramic layer and the metal coping is the chipping of the ceramic layer, thus exposing the metal surface, which compromises the aesthetics. Hence, this leads to the introduction of zirconia-based restorations in dentistry. However, even zirconium coping has the common complication of delamination or porcelain chipping from the zirconium core. Hence, the shear bond strength between the commonly used core materials and ceramic requires investigation to facilitate the materials in clinical use for longevity. Therefore, this study was conducted to compare the shear bond strength between different core materials and ceramic layering to find out the best core material for ceramic bonding.

Materials and methods

A total number of 45 samples were made as per ISO standardization (base 5 mm diameter and 1 mm thickness, step with 4 mm diameter and 4 mm in length). These samples were divided into three groups, Group A: Nickel-chromium, Group B: Cobalt-chromium, and Group C: Zirconium. Ceramic layering was layered on the top surface of each sample until an ideal height of 4 mm was obtained, and it was subjected to shear bond strength using a universal testing machine with a 50-KN load cell. This was followed by analyzing the nature of the fracture pattern using scanning electron microscopy (SEM).

Results

There were no significant differences found for the shear bond strength among group A and group B. The zirconium (group C), however, had significantly lower values than both group A and group B. The microscopic examination also revealed that the failure between the coping and the ceramic layer primarily occurred near the interface with the residual veneering porcelain remaining on the core.

Conclusions

It was found that the shear bond strength of the metal-ceramic group is better than the zirconium ceramic group, however, the fracture between the copings and the ceramic layering is found to be similar for both adhesive and cohesive failure.

## Introduction

Porcelain fused to metal systems has been extensively used in fixed partial denture (FPD) for more than 40 years and still represents the gold standard. Porcelain-fused metal systems have the combined effect of the fracture resistance of the metal substructure and the esthetics property of porcelain [1-2]. Until the early 1970s, base metals were used for removable prostheses and gold-based alloys with more than 70 weight percentage (wt. %) of pure gold were used for fixed dental prostheses. For the same volume, base metal alloys weigh less than gold, enabling the production of light and thin prostheses [3-5]. Nickel-chromium (Ni-Cr), titanium (Ti), and cobalt-chromium (Co-Cr) alloys are typically used in base metal-ceramic restoration [6]. De Melo et al. [7] investigated the bond strengths of dental ceramic to Ni-Cr alloy and Co-Cr alloy by using a shear force test and found no significant differences between the two alloys. Joias et al. [8] found that the bond strength of ceramic to a Co-Cr alloy depends on the alloy composition. In clinical scenarios, shear bond strength greater than 25 MPa is considered acceptable [9]. Even if metal-ceramic restorations are well designed, they are susceptible to porcelain fractures occurring inside the substrate or at the metal-ceramic interface.

 Hence, the introduction of partially stabilized yttria tetragonal zirconia polycrystals demonstrates the transformation toughening mechanism, with a flexural strength of 900 - 1200 MPa and a fracture toughness of 9 - 10 MPa∙m1/2. Due to its mechanical properties, zirconia has enough strength to withstand high occlusal stress [10-11]. However, delamination or a minor chip-off fracture of veneering porcelain is the frequent reason for the failures of zirconia FPDs. Therefore, the bond between the core and veneer or within the veneer material itself is one of the weaknesses in layered zirconia-based restorations and plays a significant role in their long-term success [12-15]. Thus, the shear bond strength between the commonly used core materials and ceramic requires investigation to facilitate the materials in clinical use. While Deepak et al. examined the shear bond strength of ceramic bonded to two base metal alloys that are surface treated, [16] this present study included two base metal alloys along with zirconium without any surface treatment.

## Materials and methods

**Figure 1:**
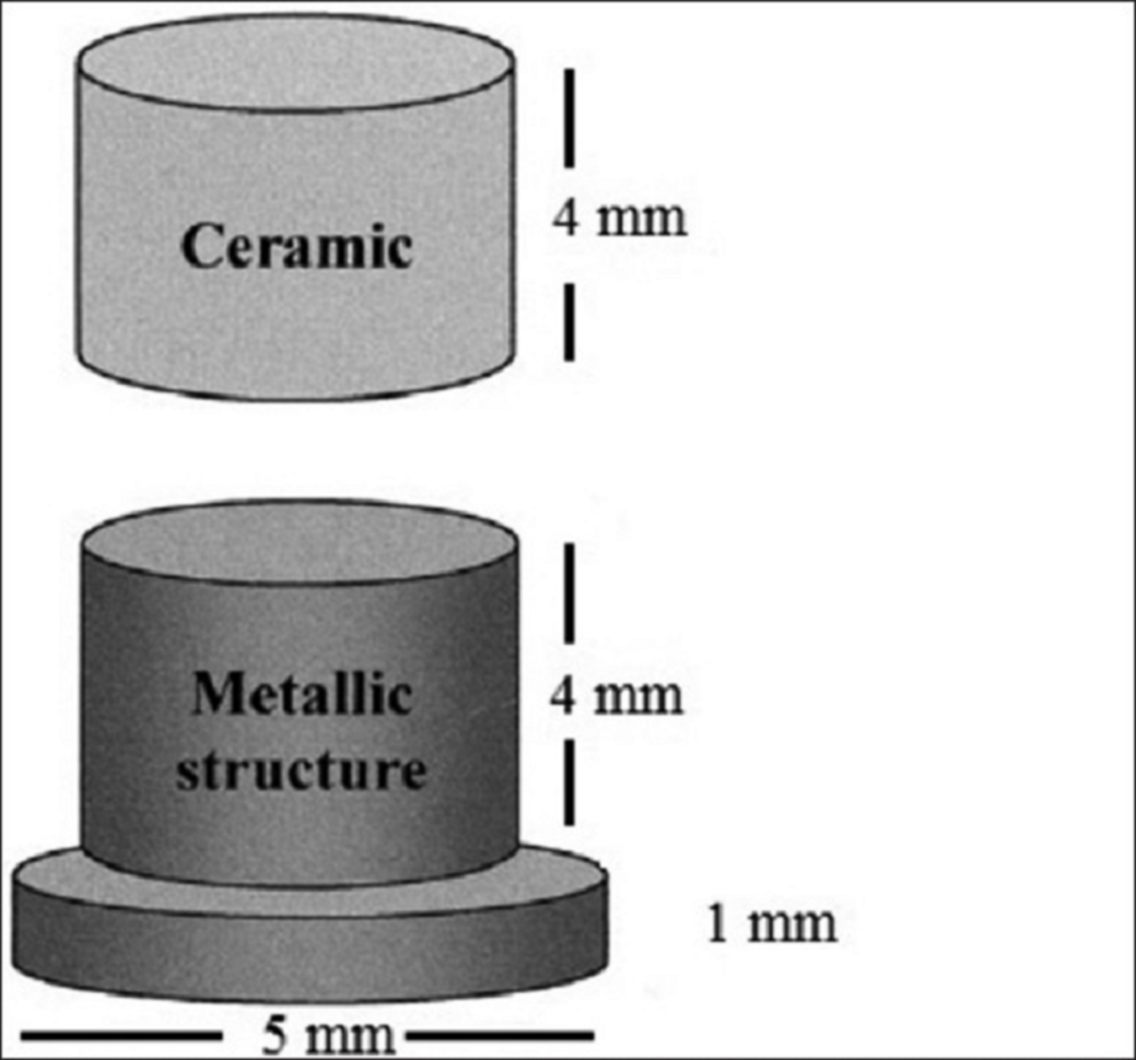
Line diagram

**Figure 2:**
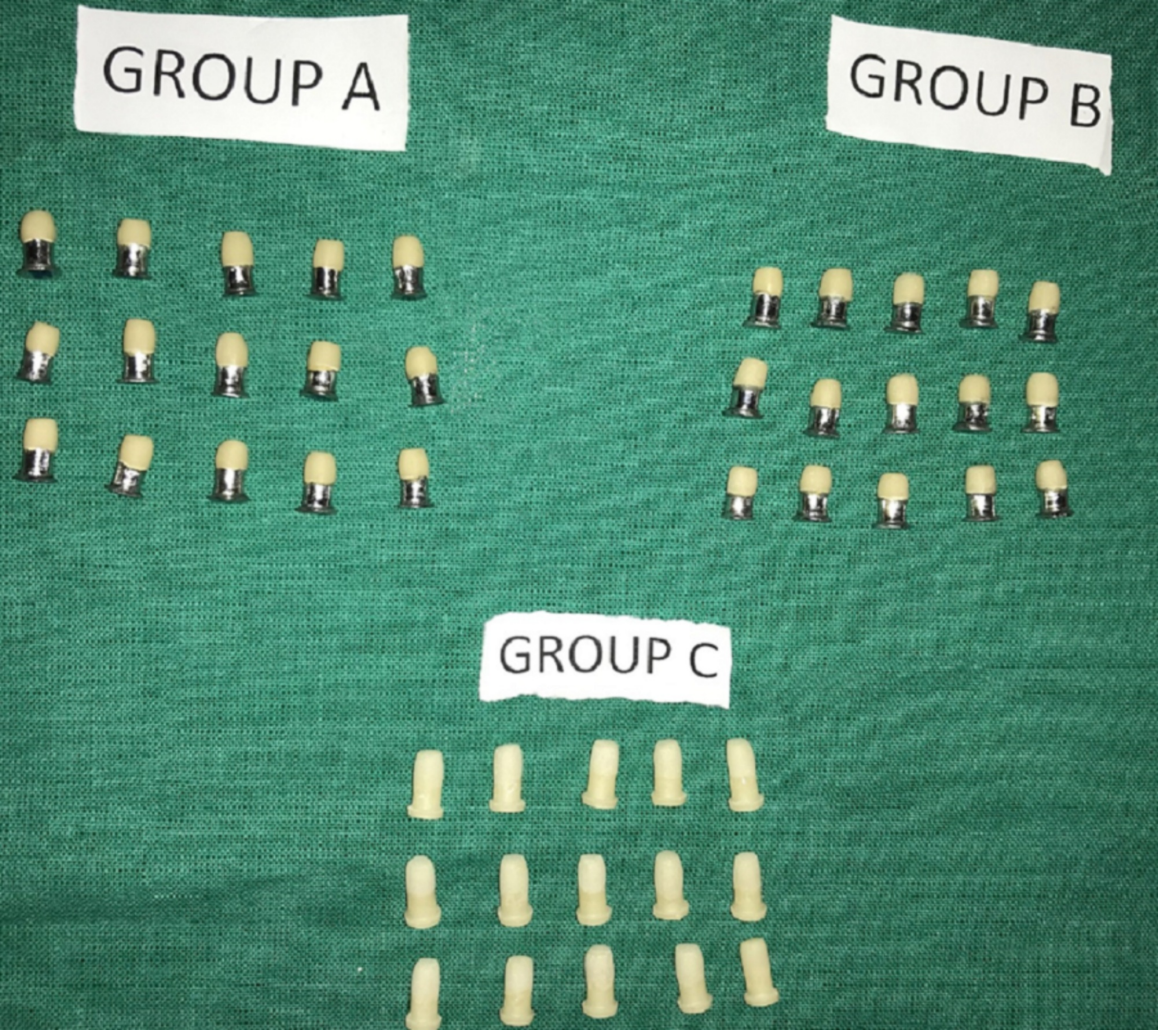
A) Ni-Cr B) Co-Cr C) Zirconium

**Figure 3:**
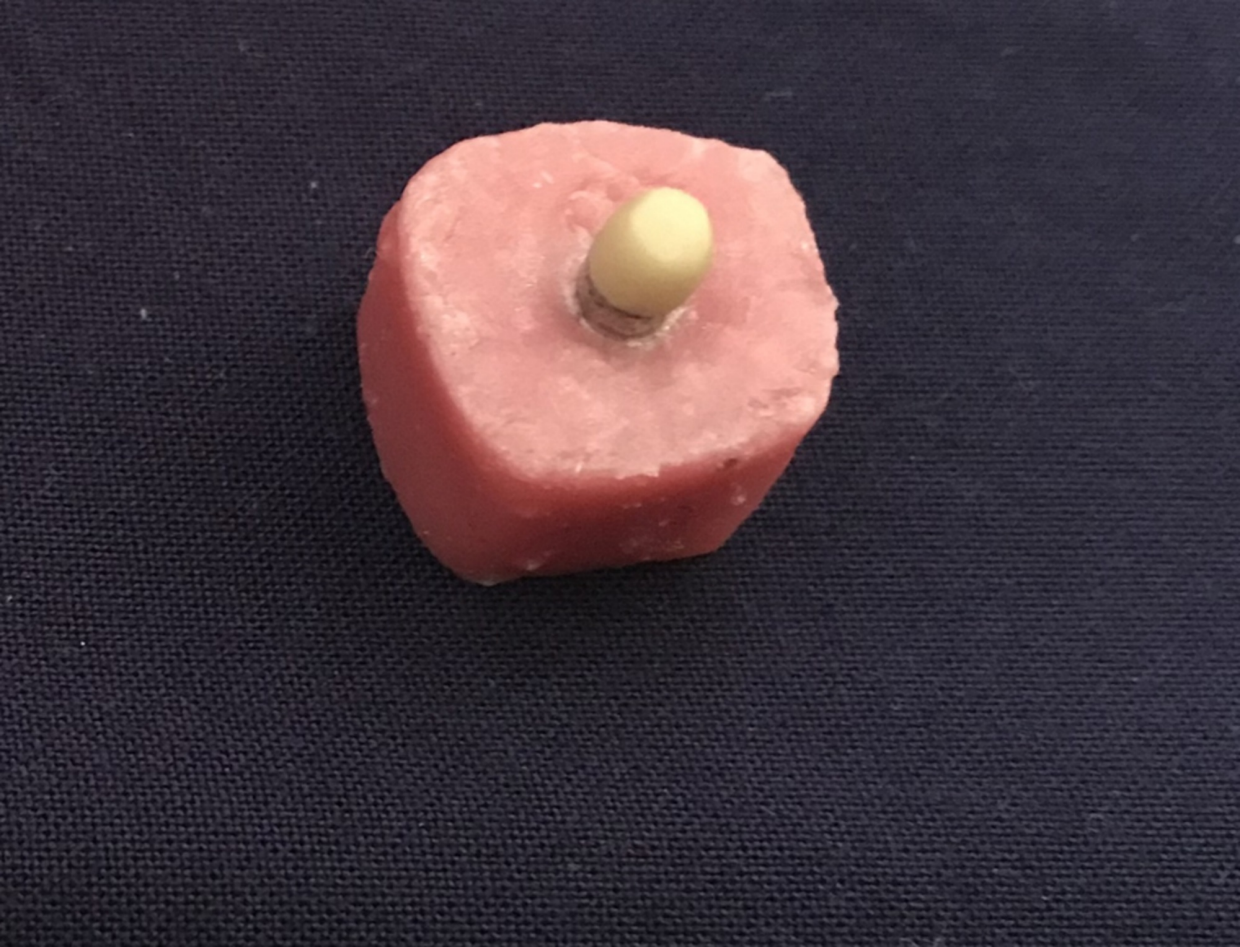
Specimen inserted in an acrylic block

**Figure 4:**
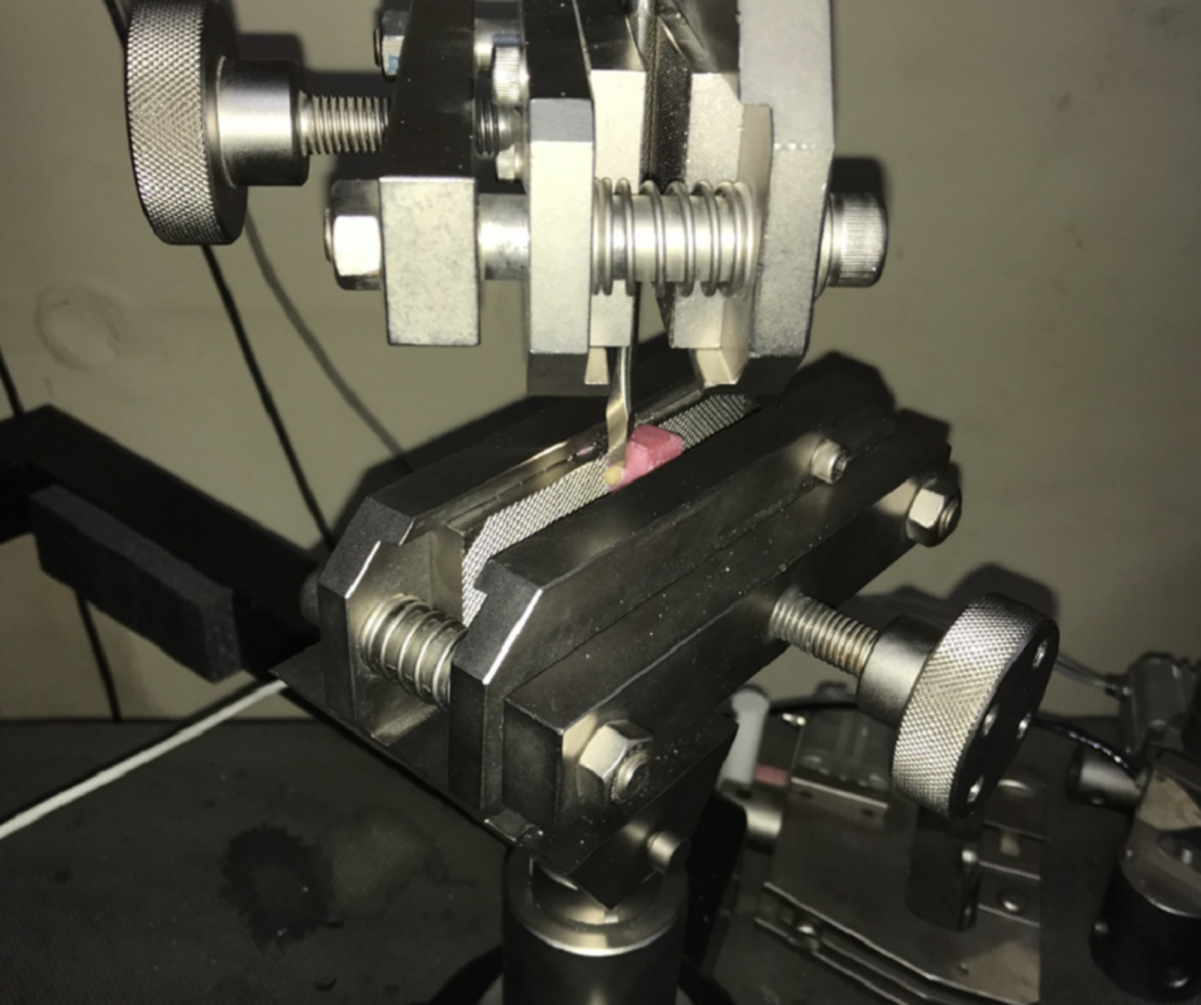
Placing the tool 1 mm above the junction Dak Universal Testing Machine (UTM) Model 1500; Dak System Inc., Mumbai, India

Three core materials, Ni-Cr (group A) and Co-Cr alloys (Group B) (Ceralloy NI, Dentalloy International Private Ltd, India.), and zirconium (Group C) (NexxZr-T- Sagemax, Bioceramics, Sweden) were used for the fabrication of 45 samples, with each group having 15 samples. The samples were fabricated according to the International Organization for Standardization (ISO). The specification of the samples is as follows: the base is 5 mm in diameter and 1 mm in thickness and the step is 4 mm in diameter and 4 mm in length [16-17] (Figure 1). For all the specimens, degassing was done to form the oxide layer, which helps in increasing the ceramic bonding. According to the manufacturer's instruction, all the 45 samples were first coated with two thin layers of opaque paste on the 4 mm diameter surface, and then the first firing was done in the ceramic furnace, followed by the application of dentin to obtain 4 mm height [16]. These samples, with a Ni-Cr, Co-Cr, and Zirconium core bonded to ceramic (Figure 2), were inserted into an acrylic block (12 mm × 12 mm × 12 mm) to the level just below the junction of the core material and ceramic (Figure 3). These samples were then subjected to shear bond testing using a Universal Testing Machine (Model 1500, Dak series; Dak System Inc., Mumbai, India) with a 50-KN load cell and a crosshead speed of 1.0 mm/min and the tool placed 1 mm above the junction until fracture occurred. Force was applied to the sample so that the shear load was exerted adjacent to the bonding interface (Figure 4). Load deflection curves and ultimate load to failure were recorded automatically and displayed by the software in the testing machine. The shear bond test was calculated as follows:

Shear stress (MPa) = Load (N) ÷ Area (mm2)

After a fracture, a scanning electron microscopy (SEM) analysis was performed to evaluate the nature of the fractured surfaces and determine whether these were adhesive or cohesive failures.

## Results

**Figure 5:**
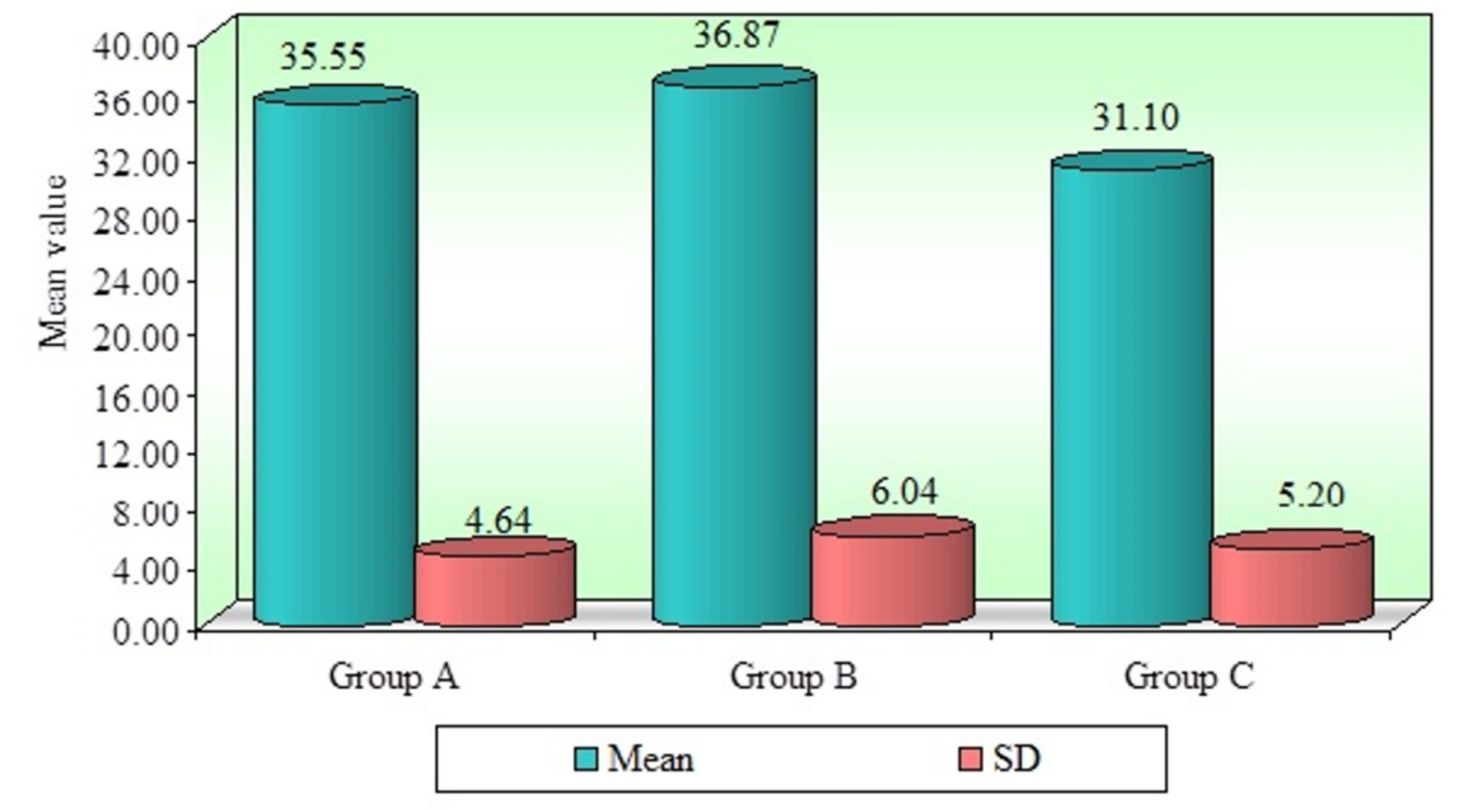
Comparison of the mean shear bond strength in the three study groups (A, B, C)

The results obtained were statistically analyzed using a pairwise post-hoc test performed using the Tukey's test for multiple comparisons. The minimum, maximum, mean bond strength, and standard deviation of shear bond strength values between the three groups are given in Table 1 and Figure 5. The mean shear bond strength and the standard deviation of ceramic bonded to Ni-Cr, Co-Cr, and zirconium are 35.55±4.64 M Pa, 36.87± 6.04 M Pa, and 31.10±5.20 M Pa, respectively. The above values indicate that Ni-Cr and Co-Cr bond strengths are nearly equal, whereas zirconium has a lower bond strength than the other two groups. 

**Table 1:** Summary of shear bond strength in three study groups (A, B, C)

Groups	Min	Max	Mean	SD	SE	95% CI for Mean	
						Lower Bound	Upper Bound
Group A	29.46	43.01	35.55	4.64	1.20	32.98	38.12
Group B	26.66	46.39	36.87	6.04	1.56	33.52	40.22
Group C	21.19	39.25	31.10	5.20	1.34	28.23	33.98

**Figure 6:**
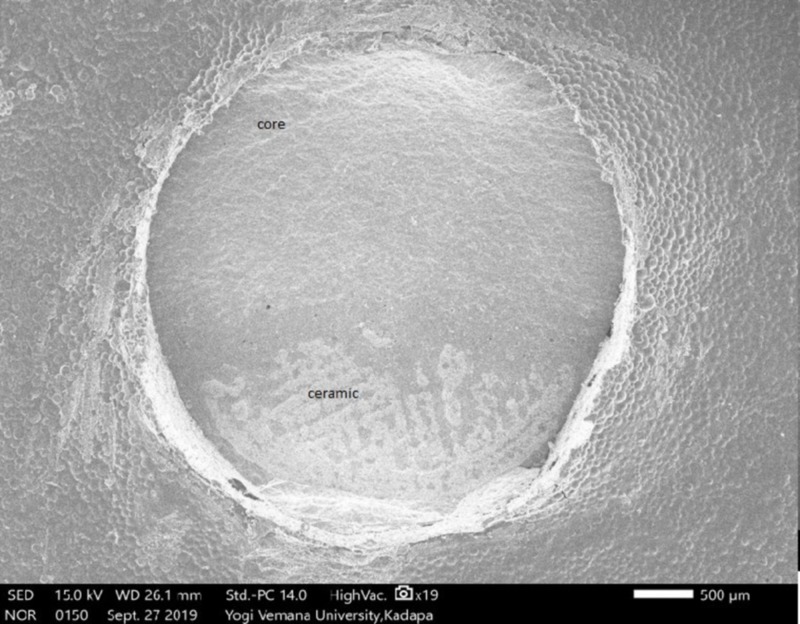
Scanning electron microscope (SEM) analysis of Ni-Cr fractured surface showing mixed failure

**Figure 7:**
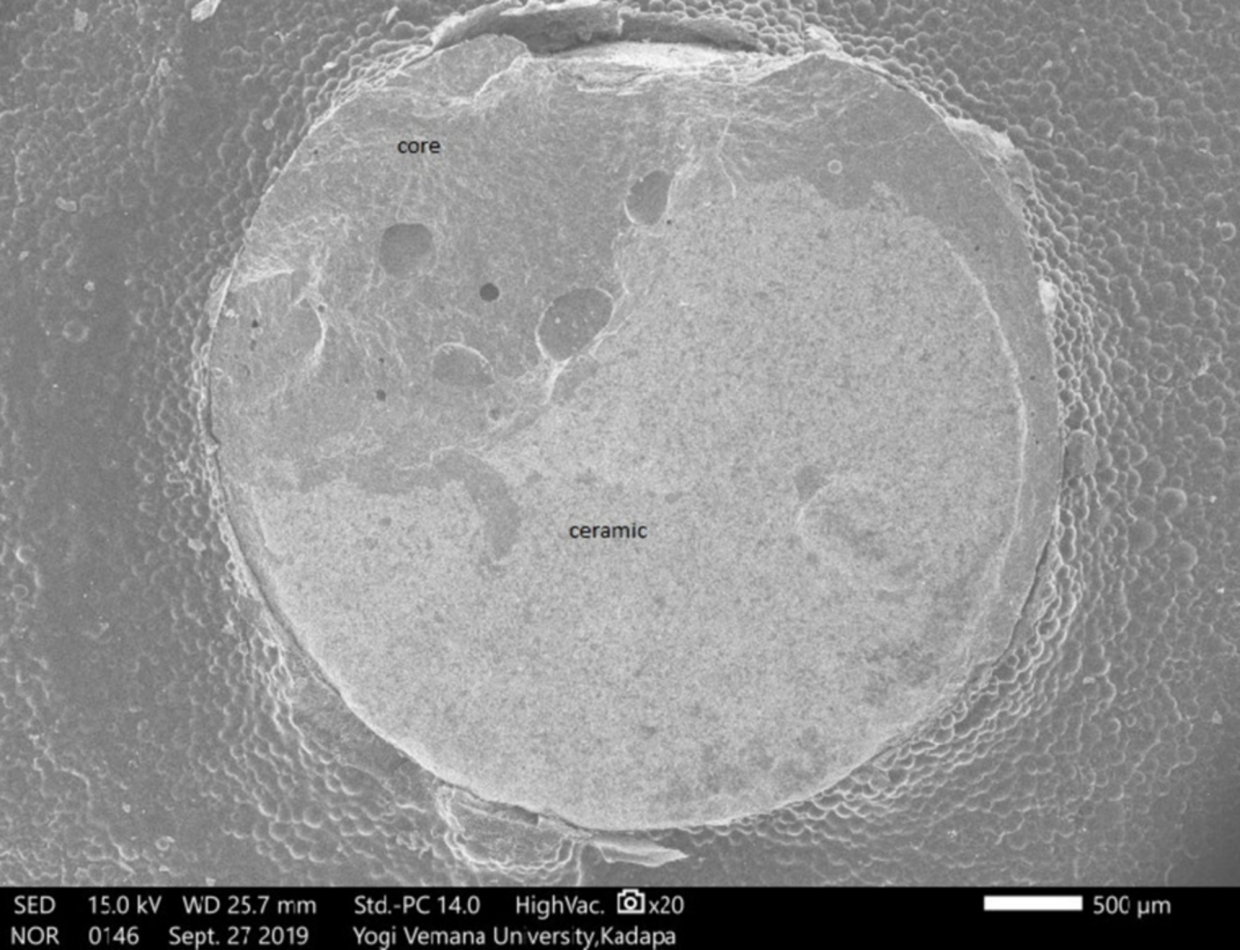
Scanning electron microscope (SEM) analysis of Co-Cr fractured surface showing mixed failure

**Figure 8:**
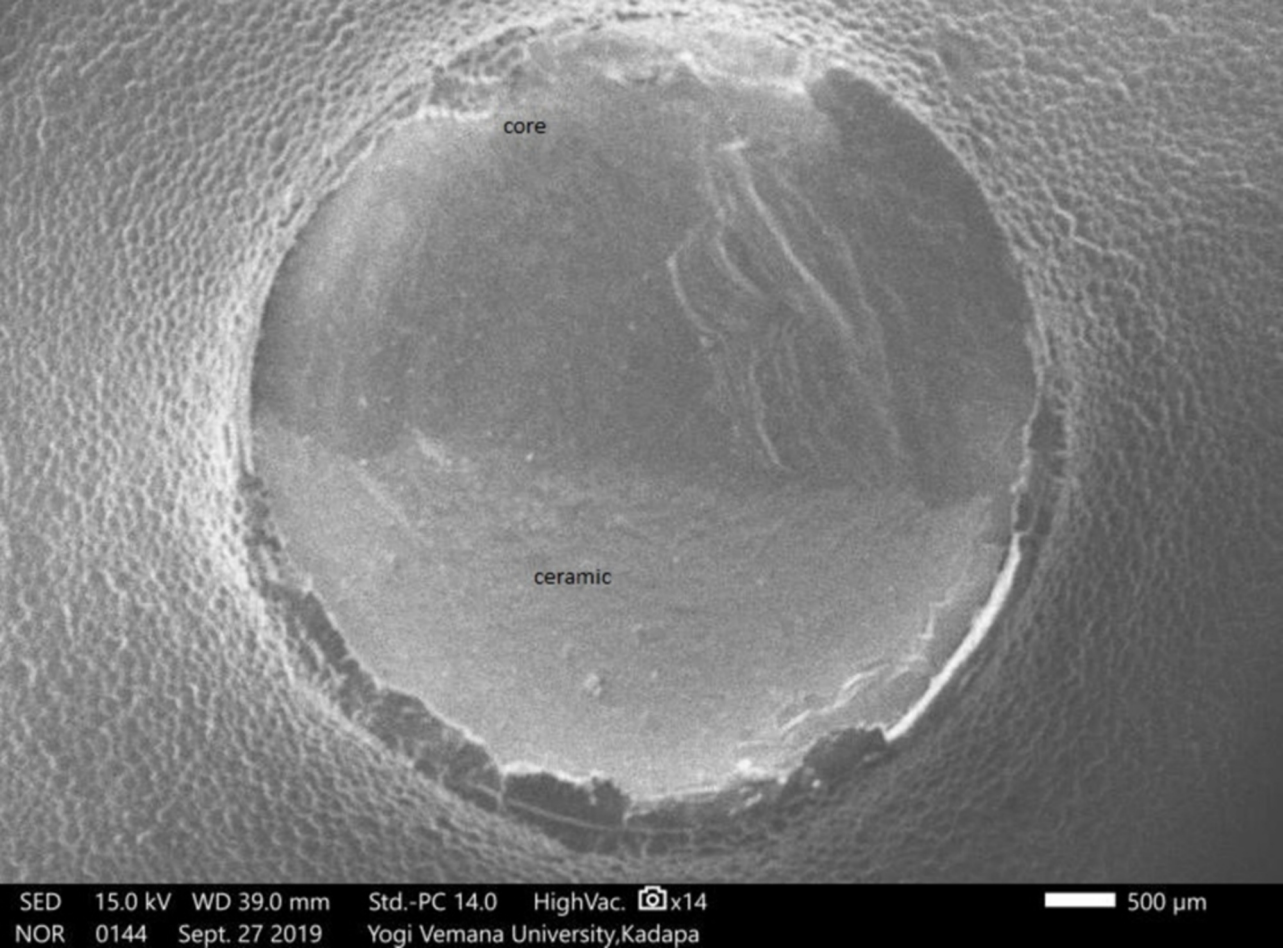
Scanning electron microscope (SEM) analysis of zirconium fractured surface showing mixed failure

Tuckey's test for post-hoc significant differences between the Ni-Cr and Co-Cr alloys was greater than .05 (P = .7770), indicating no significant difference. A comparison between Ni-Cr and zirconium was also greater than .05 (P=.069), indicating no significant difference. For the difference between Co-Cr and zirconium, a value below .05 was found (P=.01), indicating a significant difference (Table 2). The results of the SEM analysis revealed that the majority of the failures that occurred (80%) were of a mixed type (cohesive and adhesive) in Ni-Cr (Figure 6), Co-Cr (Figure 7), and zirconium (Figure 8). An SEM image of the base metal alloys and the zirconium group under high magnification (original magnification X250) showed many small pores in the veneering porcelain from which the fractures originated and propagated into the veneering ceramics. A careful examination found a thin layer of veneering porcelain covering the fracture surface.

**Table 2:** Pair-wise comparison of three study groups (A, B, C) with mean shear bond strength by Tuckey’s multiple post-hoc procedures

(I) Groups vs	(J) Group	Mean Difference (I-J)	SE	P-value	95% CI	
					Lower Bound	Upper Bound
Group A vs	Group B	-1.32	1.94	0.7770	-6.04	3.41
Group A vs	Group C	4.44	1.94	0.0690	-0.28	9.17
Group B vs	Group C	5.76	1.94	0.0140*	1.04	10.49

## Discussion

The bond strength measurement of the metal-ceramic system was standardized using the Shewickerath crack initiation test (three points bending test). To meet the ISO requirements, the mean de-bonding strength/crack initiation strength should be greater than 25 M Pa for the metal-ceramic system [2,17-18]. In this study, the shear bond strength test method was selected because of its simplicity and high reliability.

In previous studies, Dundar et al. [19] reported a shear bond strength in the range of 23-41 M Pa, and Al-Dohan reported shear bond strength in the range of 22-31 M Pa for commercially available core-veneer all-ceramic systems (zirconium). In this study, the shear bond strength (SBS) value of veneering ceramic to a zirconium core was 31.10 M Pa, confirming the finding of previous studies. However, unlike in the Al-Dohan study [20], our study results indicate a significant difference in mean SBS value between the zirconium group and the metal group. This difference in findings could be attributed to many factors, such as study design, methodology, skill, experience with the apparatus, and different properties of different materials. Some evaluations revealed that the fracture originated in the veneering porcelain in both the zirconium and metal-ceramic groups. The failure modes from the metal-ceramic and zirconium groups suggest the importance of the mechanical properties of veneering porcelain, as cracks initiated in the veneering porcelain. It is possible that internal defects of the veneer led to the initiation of fracture; thus, the fabrication techniques, such as layering, firing, surface finishing, and polishing of veneering porcelain, are critical [21]. Besides, the strength of the veneering porcelain is also related to the degree of crystallinity, paramount to the longevity of the restorations [22]. This study was performed in a dry environment without the influence of saliva, temperature, and pH changes [23]. Therefore, thermocycling or artificial aging procedures should be included in subsequent studies.

## Conclusions

Within the limitations of this study, we concluded that the metal-ceramic group had more shear bond strength than zirconium, and both metal-ceramic groups have similar strength. The SEM analysis indicated that the three groups have combined failure modes that originated in the veneering porcelain in both the zirconium and metal groups. The fracture origin in the veneering porcelain was mostly on the loaded surface. However, more studies have to be done by using an ample number of samples. In addition, the samples should be subjected to the tests in the clinical scenario to provide more reliability and accuracy.
